# ^18^F-FDG-PET/CT in breast cancer imaging: Restaging and Implications for treatment decisions in a clinical practice setting

**DOI:** 10.2340/1651-226X.2024.40003

**Published:** 2024-08-11

**Authors:** Ida Skarping

**Affiliations:** a Division of Oncology, Department of Clinical Sciences, Lund University, Skåne University Hospital, Lund, Sweden; b Department of Clinical Physiology and Nuclear Medicine, Skane University Hospital, Lund, Sweden

**Keywords:** Breast cancer, imaging, staging, ^18^F-FDG, PET/CT, clinical management

## Abstract

**Background and purpose:**

Although the diagnostic accuracy of ^18^F-fluorodeoxyglucose – positron emission tomography/computed tomography (^18^F-FDG-PET/CT) for breast cancer (BC) has been well studied, few studies have evaluated the impact of ^18^F-FDG-PET/CT on BC patient care. This study aimed to investigate restaging and ^18^F-FDG-PET/CT-induced changes in clinical decision-making in patients with BC.

**Material and methods:**

We retrospectively evaluated ^18^F-FDG-PET/CT-scans performed for BC-related indications in a prospectively collected consecutive cohort of adult patients at Skane University Hospital, Sweden. Patients with all BC stages were included and divided into three groups based on the indication for ^18^F-FDG-PET/CT: Group A (primary staging), Group B (response evaluation), and Group C (recurrence). The impact of ^18^F-FDG-PET/CT-scans on clinical management was categorized as no change, minor change (e.g. modification of treatment plans), or major change (e.g. shift from curative to palliative treatment intention).

**Results:**

A total of 376 scans (151 patients) were included: Group A 9.3% (35 of 376 scans), Group B 77.4% (291 of 376 scans), and Group C 13.3% (50 of 376 scans). Significant stage migration, predominantly upstaging, occurred in Group A (45.7%) and Group C (28.0%). Changes in clinical management were observed in 120 scans (31.9%), of which 66 were major and 54 were minor. The largest proportion of ^18^F-FDG-PET/CT-induced management changes were observed in Group A (57.1%), most commonly a shift from curative to palliative treatment intention due to upstaging.

**Interpretation:**

Our study indicates the clinical utility of ^18^F-FDG-PET/CT in BC restaging and changes in clinical management; the latter observed in approximately one-third of all cases.

## ^18^F-FDG-PET/CT in breast cancer imaging: Restaging and Implications for treatment decisions in a clinical practice setting

**Figure:**
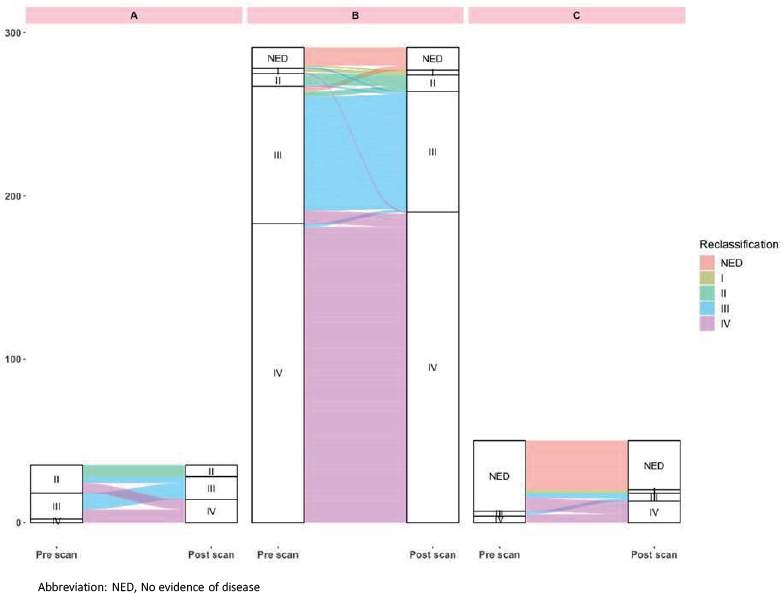

Change in clinical managment

**Figure:**
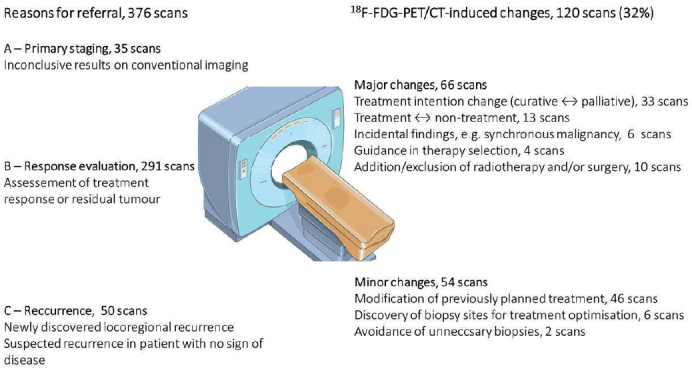

## Introduction

Breast cancer (BC) stage is an important prognostic factor for recurrence and overall survival [1]. Imaging is used to guide clinical decision-making and optimize treatment strategies. Therefore, the choice of diagnostic modality is important. While mammography, ultrasound, and magnetic resonance imaging (MRI), are used for imaging of the breast and axilla, contrast-enhanced computed tomography (i.e. CT) is used widely for staging here beyond. When applicable, bone scintigraphy can be used to detect recurrence [2]. However, positron emission tomography (PET) is a valuable instrument for comprehensive whole-body imaging in BC management in both the initial staging and metastatic setting [3–5].

Contrary to visualizing anatomical structures, PET is a functional imaging modality used to visualize the uptake of radioactive substances, for example, ^18^F-fluorodeoxyglucose (^18^F-FDG). PET is combined with CT in PET/CT, where the CT findings act as an anatomical reference for the molecular and functional information provided by PET, in addition to attenuation correction [6]. Following ^18^F-FDG administration, accumulation in tissue is proportional to the degree of glucose metabolism, where high metabolic activity is one of the hallmarks of cancer, including BC [7,8]. Although the uptake mechanism is similar to that of glucose in cells, the radiotracer is intracellularly trapped and thus not fully metabolized [6], which is a prerequisite for ^18^F-FDG-PET diagnostics.

The diagnostic accuracy of detecting distant BC metastases with ^18^F-fluorodeoxyglucose – Positron emission tomography/Computed tomography (^18^F-FDG-PET/CT) is high; in a meta-analysis by Hong et al. [9], the pooled sensitivity was 96% and the negative likelihood ratio was 0.03. As demonstrated by Koolen et al., for regional BC metastatic evaluation, the sensitivity of ^18^F-FDG-PET/CT is lower at 82%, however, still with a high specificity of 92% [10]. Moreover, accurate BC staging with ^18^F-FDG-PET/CT varies with tumor characteristics including St Gallen surrogate and histological subtypes [4,11,12]; a lower sensitivity of ^18^F-FDG-PET is seen in lobular BC due to its more indolent biology and lower ^18^F-FDG avidity [4,11,12]. Considering another PET radiotracer in BC, the recently updated NCCN guidelines now include ^18^F-Fluorestradiol (^18^F-FES) PET for potential use in evaluating estrogen receptor-positive metastatic BC [13].

^18^F-FDG-PET/CT is typically indicated only for initial staging in patients with stage IIB-III BC when distant metastases are suspected [13,14]. Additionally, unclear or contradictory findings on conventional imaging or specific symptoms may lead to a referral for ^18^F-FDG-PET/CT regardless of stage [13,15,16]. For patients with stage I–II and operable stage III (T3, N1), routine use of ^18^F-FDG-PET/CT is not recommended due to high false-negative rates for small lesions and a low overall probability of metastatic disease [13,15]. ^18^F-FDG-PET/CT may also be beneficial for treatment evaluation in both neoadjuvant and metastatic settings [13,14], especially for monitoring bone-only/-predominant metastases [17,18] (Supplementary Material 1).

While diagnostic accuracy is of paramount importance, in a clinical context, scan-induced restaging and modification of treatment are also highly relevant. Although the ultimate endpoint is a gain or loss in morbidity and mortality, the direct clinical consequences of diagnostics can be less obvious than those of a therapeutic procedure [19]. The concept of clinical utility emphasizes that a diagnostic test’s health benefit arises from using information to guide management or resolve uncertainty, reducing patients’ emotional burden [19].

This study aimed to investigate stage migration and changes in clinical management following ^18^F-FDG-PET/CT-scan in a heterogeneous consecutive cohort of clinical BC patients.

## Material and methods

### Cohort data

Eligible participants in this retrospective analysis included adult patients (aged ≥ 18 years) with a clinical indication for ^18^F-FDG-PET/CT due to BC at Skane University Hospital, Sweden, between November 2017 and October 2023, who were prospectively included in a larger, overarching institution-based study for validation of PET/CT.

**Figure 1:**
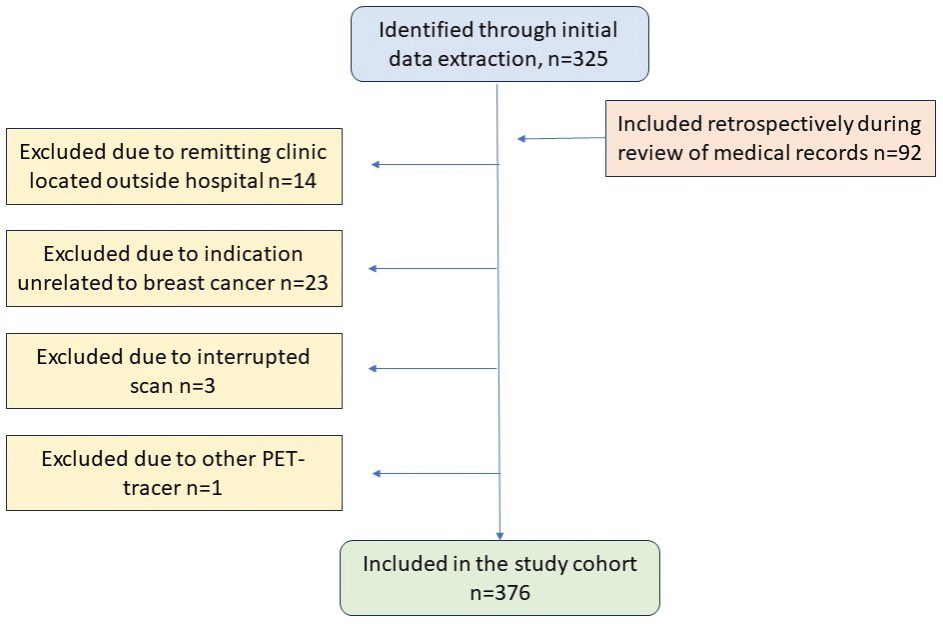
Flow chart of study cohort.

Initially, 325 consecutive scans were identified. Additional 92 ^18^F-FDG-PET/CT scans performed on patients in the cohort, but not previously identified due to missing/inaccurate annotation in the validation study, were also included. A total number of 41 scans were excluded (Figure 1).

Demographic data, blood glucose level, and reason for medical referral were obtained from the validation study for all patients at each scan. Clinicopathological data, oncological treatment, and Tumor Node Metastasis (TNM) stage before and after the ^18^F-FDG-PET/CT-scan were verified by reviewing the patient’s digital medical record and picture archiving and communication system.

This study was designed and the manuscript was written in accordance with the STROBE guidelines [20].

### Definitions

The TNM stage was determined using the guidelines established by the American Joint Committee on Cancer [21] in cases where the TNM stage was not explicitly stated in the medical records. The TNM stage was determined through the assessment of all available imaging and biopsy results. The classification of ‘no evidence of disease’ was defined as complete remission pathologically/metabolically and structurally for Group B and C.

### ^18^F-FDG-PET/CT

^18^F-FDG-PET/CT scans were performed using Discovery MI or Discovery 690 (GE Healthcare, Milwaukee, USA) PET/CT system. ^18^F-FDG-radiotracers were prepared according to established techniques and clinical routines. The intravenously administered activity was 4 MBq/kg (maximum 500 MBq). Approximately 60 minutes after injection, the scan was performed in the supine position with arms raised (unless mobility was restricted). As per standard procedure, the scan covered the area from the orbitomeatal line to the upper thigh, with an acquisition time of approximately 1.5 minutes per bed position. Attenuation correction and anatomic correlation were achieved through a low-dose CT or diagnostic CT (with contrast if there were no contraindications).

### Clinical management: Group allocation into three categories

**Figure 2:**
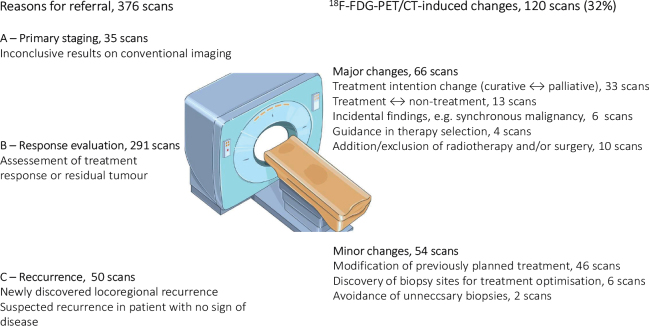
Schematic figure: reason for referral and scan-induced changes in clinical management.

The scans were divided into three groups based on the reasons for ^18^F-FDG-PET/CT referral: Group A, Primary staging (unclear/contradictory findings in conventional imaging scans); Group B, Response evaluation (assessment of treatment response/residual tumor); and Group C, Recurrence (suspected recurrence or proven locoregional recurrence) (Figure 2). All newly diagnosed patients were allocated to Group A. While the patients in Group B and Group C might overlap, those categorized as C had no ongoing treatments and were considered to be in remission or had discovered locoregional recurrence without current knowledge of distant spread.

^18^F-FDG-PET/CT-induced management changes were divided into three prespecified categories: no change, minor change (e.g. modification of treatment), and major changes (e.g. change of treatment) (Figure 2).

### Statistical analyses

Categorical variables were summarized as counts/percentages and continuous variables as medians/interquartile ranges (IQRs). Statistics are presented both per-scan and per-patient due to a substantial number of patients having multiple scans. Demographics were analysed using chi-square tests for categorical variables and Kruskal–Wallis test for continuous variables. For assessment of reclassification in Group A, B, and C, respectively, Wilcoxon signed ranks test was used.

A *p* value < 0.05 was considered significant. No *a priori* power calculations were performed for this consecutive cohort.

IBM SPSS Statistics version 28 was used for all statistical analyses.

## Results

### Patient and tumor characteristics: Descriptive results

The patient characteristics of the 376 scans (151 individual patients) are described in Table 1, Supplementary Material 2. The median age was 61 years. A total of 79 patients had multiple scans performed (range 2–18) and 72 patients had single scans performed. The distribution of the tumor characteristics estrogen receptor, progesterone receptor, human epidermal growth factor receptor 2, and histological subtypes were similar in Group A–C, whereas highly proliferative tumors (Ki67) were more common in Group A (Table 1). Invasive BC of no special type (NST) BC was the most common histological subtype in all groups (range 63–76%) followed by ILC (range 12–21%) (Supplementary Material 3). Previous and ongoing treatment per person is outlined in Supplementary Material 4.

**Table 1:** Cohort characteristics, pre-scan: patients’ and tumor characteristics at time of referral to ^18^F-FDG-PET/CT, per-scan analysis.

Scans, 376 scans		Group A				Group B				Group C				*p*
		*n*	%	Median	IQR	*n*	%	Median	IQR	*n*	%	Median	IQR	
Total scans (%) (NB: Row percent)		35	9.3			291	77.4			50	13.3			
Age at each scan (years)	Median (IQR)			61	48–71			61	49–71			58	45–72	0.416
BMI (kg/m^2^)	Median (IQR)			25.2	22.1–28.5			25.8	22.1–28.5			26.0	22.7–29.8	0.784
	Missing (*n*)	2				21				3				
Blood glucose (mmol/L)	Median (IQR)			5.6	5.1–6.3			5.5	5.1–6.0			5.6	5.0–6.1	0.701
	Missing (*n*)	4				26				4				
Estrogen receptor status^a,b^	Positive	25	72.2			229	79.2			32	74.4			0.614
	Negative	9	26.5			60	20.8			11	25.6			
	Missing	1				2				7				
Progesterone receptor status^a,b^	Positive	24	72.7			186	64.8			29	67.4			0.644
	Negative	9	27.3			101	35.2			14	32.6			
	Missing	2				4				7				
HER2 status^a,c^	Positive	6	18.8			31	10.9			39	92.9			0.277
	Negative	26	81.3			254	89.1			3	7.1			
	Missing	3				6				8				
Ki67^a^	> 20% (high)	27	84.4			127	59.6			25	71.4			0.015*
	≤ 20% (low)	5	15.6			86	40.4			10	28.6			
	Missing	3				78				15				
Pre-scan TNM stage	No evidence of disease	0				13	4.5			43	86.0			
	I	0				3	1.0			0				
	II	17	48.6			8	2.7			0				
	III	16	45.7			84	28.9			3	6.0			
	IV	2	5.7			183	62.9			4	8.0			

Chi-square test for categorical variables and Kruskal Wallis for continuous variables.

BMI: body mass index; ^18^F-FDG-PET/CT: ^18^F-fluorodeoxyglucose – positron emission tomography/computed tomography; HER2: human epidermal growth factor receptor 2; TNM: tumor node metastases.

a Tumor characteristics based on biopsy results from the primary tumor at the time of breast cancer diagnosis. In the cases of missing data on the primary tumor, the latest biopsy result was used.

b Positive expression of hormone receptors was defined as ≥ 10% for each receptor [15].

c HER2-amplification was defined as 2 + and FISH-positive, or 3 +.

* *p* < 0.05.

### Stage migration

**Figure 3:**
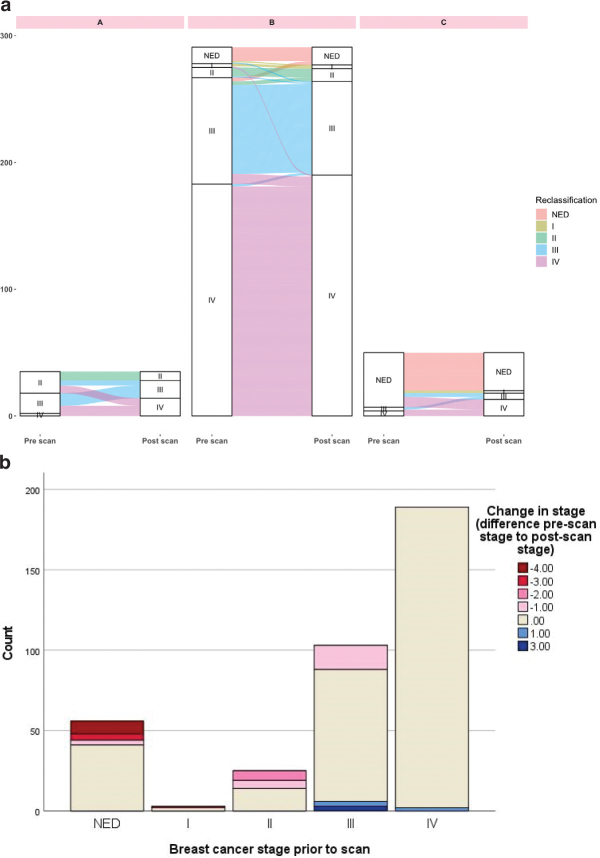
(A) Sankey diagram visualizing changes in breast cancer stage in each Group A–C. The Sankey diagram was made using R version 4.2.2. (B) Restaging according to stage prior to scan. -4 to -1 indicates upstaging, 0 equals no change in stage, and 1–3 indicates downstaging.

In total, 13.3% (50 of 376) of the scans resulted in restaging, most commonly upstaging (42 of 50, 84%) (Tables 2, 3, and Figure 3). A significant change in stage was observed in Groups A and C but not in Group B (Table 2). All eight scans resulting in downstaging occurred in Group B (response evaluation) (Table 3, Figure 3). In total, 15 of 56 scans (26.8%) categorized as having no evidence of disease pre-scan were restaged. A larger proportion of restaging was observed in the earlier BC stages: 42.9% (12 of 28) in stage I–11, 20.4% (21 of 103) in stage III, and 1.1% (2 of 189) in stage IV (downstaging) (Supplementary Material 5).

**Table 2:** TNM stage prior and after ^18^F-FDG-PET/CT scan, per-scan analysis, *n* (%).

Group	Stage	Before ^18^F-FDG-PET/CT		After ^18^F-FDG-PET/CT		*p*
		*n*	%	*n*	%	
A, Primary staging (35 scans)	I	0	0	0	0	< 0.001*
	II	17	48.6	7	20.0	
	III	16	45.7	14	40.0	
	IV	2	5.7	14	40.0	
B, Response evaluation (291 scans)	NED	13	4.5	14	4.8	0.665
	I	3	1.0	3	1.0	
	II	8	2.7	10	3.5	
	III	84	28.9	74	25.4	
	IV	183	62.9	190	65.3	
C, Recurrence (50 scans)	NED	43	86.0	30	60.0	< 0.001*
	I	0	0	2	4.0	
	II	0	0	0	0	
	III	3	6.0	5	10.0	
	IV	4	8.0	13	26.0	

TNM: tumor node metastases; ^18^F-FDG-PET/CT: ^18^F-fluorodeoxyglucose – positron emission tomography/computed tomography; NED: no evidence of disease.

Wilcoxon Signed Ranks Test,

* *p* < 0.05.

**Table 3:** Change in stage, difference between stage prior to and after ^18^F-FDG-PET/CT-scan, stratified by indication.

Change in stage (difference between stage pre-scan and post-scan)	-4	-3	-2	-1	0	1	2	3	4	Total restaging
A, Primary staging (35 scans)	0	0	6	10	19	0	0	0	0	16 (45.7%)
B, Response evaluation (291 scans)	0	2	0	10	271	5	0	3	0	20 (6.9%)
C, Recurrence (50 scans)	8	3	0	3	36	0	0	0	0	14 (28.0%)
Total, 376 scans	8	5	6	23	326	5	0	3	0	50 (13.3%)
										*p* < 0.001*

^18^F-FDG-PET/CT: ^18^F-fluorodeoxyglucose – positron emission tomography/computed tomography.

* The two-tailed Chi-square test

In Group B, analysis of stage migration on a a per-patient basis showed a larger proportion of restaging compared to the per-scan analysis (12 of 76, 15.8% vs. 20 of 291, 6.9%) (Supplementary Material 6–9).

### ^18^F-FDG PET/CT-induced change in clinical management

Of the 376 scans, 120 (31.9%) led to changes in the clinical management. A total of 54 of 120 (45.0%) changes were classified as minor changes, and 66 of 120 (55.0%) were classified as major changes (Figure 2, Table 4). In 256 scans, the ^18^F-FDG-PET/CT-scan did not lead to verified changes in clinical management. The largest proportion of scan-induced changes was observed in Group A (57.1%), followed by Group C (32.0%). In both groups, the specific change was related to the findings of metastatic disease and thus re-evaluation of treatment intention.

**Table 4:** ^18^F-FDG-PET/CT scan-induced changes in clinical management, per-scan analysis.

Type of change	No change	Minor change						Major change								Total
		1	2	3	4	5	Total	1	2	3	4	5	6	7	Total	
A, Primary staging (35 scans)	15	1	0	0	2	0	3	0	15	0	0	0	2	0	17	20 (57.1%)
B, Response evaluation (291 scans)	207	1	42	5	0	2	50	3	9	1	3	6	2	10	34	84 (28.9%)
C, Recurrence (50 scans)	34	0	0	1	0	0	1	0	6	0	9	0	0	0	15	16 (32.0%)
Total, 376 scans	256	2	42	6	2	2	54	3	30	1	12	6	4	10	66	120 (31.9%)

^18^F-FDG-PET/CT: ^18^F-fluorodeoxyglucose – positron emission tomography/computed tomography.

Minor change: 1 Modified radiotherapy; 2 Modified systemic treatment; 3 Biopsy to further optimize clinical management; 4 Avoidance of unnecessary diagnostics; 5 Modified surgical plan.

Major change: 1 Downstaging and change from palliative to curative; 2 Upstaging and change from curative to palliative; 3 Complete remission, change from treatment to non-treatment; 4 Change from non-treatment to treatment; 5 Secondary findings on ^18^F-FDG-PET/CT-scan affecting clinical management (e.g. other malignancy/pathology); 6 ^18^F-FDG-PET/CT-scan guides treatment plan; 7 Addition/exclusion of surgery and/or radiotherapy to systemic treatment.

In the response evaluation Group B (291 scans), the majority of documented minor changes (50 scans) were in the form of modifications to previously planned treatments (45 scans), whereas in five scans, information on biopsy sites was determined. The major changes observed in this group (34 scans) exhibited a diverse range of alterations.

Analysis on a per-patient basis showed a larger proportion of scan-induced changes in clinical management (61 of 151, 40.4% vs. 120 of 376, 31.9%). Differences in scan-induced changes were observed in Group B (response evaluation) (32.9% vs. 28.9%) and Group C (recurrence) (40.0% vs. 32.0%) (Supplementary Material 10).

## Discussion

In this retrospective cohort study involving 376 consecutive clinical ^18^F-FDG-PET/CT scans performed on BC patients across diverse clinical settings, our findings reveal a high rate of restaging, primarily upstaging. Moreover, one-third of all scans resulted in alterations in clinical management. The most frequent changes involved modifications to the planned systemic treatment, such as adjustments to dosage or switching from one chemotherapy regimen to another, and instances in which the treatment objective was changed from curative to palliative.

Our cohort consisted primarily of patients in late stages (77.7% of the scans BC stage III–IV) prior to undergoing the scan, which aligns with the current national and international guidelines and enhances the validity of our finding [14–18]. The usefulness of ^18^F-FDG-PET/CT imaging in the late-stage setting was demonstrated by the detection of suspected recurrence in 16 of 50 scans (32.0%) conducted on patients in remission without evidence of disease. However, the highest rate of restaging and scan-induced changes in clinical management was observed in patients undergoing ^18^F-FDG-PET/CT as part of their initial work-up, who generally had a lower BC stage.

The presented data on the reclassification of staging and alterations in clinical management prompted by ^18^F-FDG-PET/CT-scans collectively suggest that ^18^F-FDG-PET/CT could serve as a versatile tool for the clinical management of BC patients across a range of clinical contexts.

### Comparison to previous literature

#### Stage migration

In their meta-analysis, Han et al. [3] reported a change in BC stage in a pooled proportion of 20 and 25% of patients who underwent PET/low-dose CT and PET/diagnostic CT, respectively, during their initial BC work-up, without any specific stage or histological subtype criteria. Groheux et al. [22] conducted a prospective study on 131 patients with stage IIA–IIIA disease and observed changes in stage of 6, 15, and 28% within their stage IIA, IIB, and IIIA groups, respectively. In contrast, our cohort showed 44.0% (11 of 25) of stage II patients and 20.3% (21 of 103) of stage III patients experiencing restaging. However, Groheux et al. did not include patients with an initial stage IV disease, which constituted the majority of our cohort. Yararbas et al. [23] conducted a retrospective study on 234 patients and detected metastases in 64 of them, corresponding to stage migration from IIA–IIIC to stage IV in 28% of the cohort. Vogsen et al. [24] investigated restaging in a Danish study cohort of 103 high-risk primary BC patients with tumor size ≥ 50 mm or ≥ 4 malignant axillary lymph nodes, where ^18^F-FDG-PET/CT detected previously unknown distant metastases in 23% (24 of 103) of the patients.

The results of our study emphasize findings from previous studies investigating stage migration after ^18^F-FDG-PET/CT-scans, the results are congruent with the general conclusion that ^18^F-FDG-PET/CT-scans precedes stage changes across various clinical BC scenarios. Nonetheless, we acknowledge that the clinical significance may be smaller in more advanced stages, such as metastatic stage IV BC. Still, it remains interesting to assess the influence of ^18^F-FDG-PET/CT scans in stage IV BC patients; for example, in treatment response evaluation, ^18^F-FDG PET/CT more often discriminates between progression/regression and stable disease compared to conventional imaging techniques [25]. Additionally, ^18^F-FDG-PET/CT offers distinct advantages over other imaging techniques, such as conventional CT, as it facilitates earlier identification of progression or response during treatment [26–28].

#### ^18^F-FDG PET/CT induced change in management

Although ^18^F-FDG-PET/CT has been repeatedly demonstrated to upstage patients [3], the clinical impact of the scan results is less often evaluated. In this study, we observed that ^18^F-FDG-PET/CT led to changes in management in 31.9% of the 376 scans performed, with approximately half of these changes considered major (66 of 120, 55.0%), most commonly change from curative to palliative treatment as a result of upstaging. The greatest proportion of scan-induced changes was observed in patients undergoing scans as part of their primary staging workup (57.1%, 20 of 35). In total, 54 changes were minor, mostly modifications to previously planned or ongoing systemic treatment. Notably, in three patients undergoing ^18^F-FDG-PET/CT as part of restaging, no metabolic activity was detected in previously suspected distant metastases, leading to a lower stage and a shift in treatment from palliative to curative.

Given the different prerequisites in each group, the scan-induced changes in Group A–C should be contextualized. Owing to the diverse nature of the cohort and the reasons for undergoing ^18^F-FDG-PET/CT scans, the significance of scan-induced changes may vary between the groups. In primary staging, ^18^F-FDG-PET/CT is often regarded as an alternative to conventional imaging (i.e. CT), due to unclear/contradictory findings. By contrast, ^18^F-FDG-PET/CT may be the preferred imaging modality for restaging and treatment assessment, with no comparable conventional imaging methods.

Our findings emphasize previous findings from the aforementioned meta-analysis by Han et al. [3], which reported clinical changes after ^18^F-FDG-PET/CT-scans in a pooled proportion of 17% of cases; our study found a higher proportion of 32%. Furthermore, a study by Vogsen et al. [24] demonstrated a substantial scan-induced impact on clinical management during primary staging in 39% of 103 patients. However, their study was limited to patients undergoing primary staging and was analyzed on a per-patient basis. In contrast, our study included all stage BC and analyzed the scans both on a scan-by-scan and patient-by-patient basis, as nearly half of the patients underwent multiple scans.

We aimed to provide a comprehensive and detailed categorization of the impact of ^18^F-FDG-PET/CT scans on clinical management, as opposed to the categorization of changes according to intermodality (alteration in the type of management) and intention-to-treat by Han et al. [3]. Vogsen et al. [24] defined changes in management as either changes in treatment or incidental findings with clinical consequences, while Yarabras et al. [23] analyzed changes in management in relation to therapy selection. Our study builds upon these previous analyses by including all previously mentioned changes in management, as well as including the avoidance of unnecessary biopsies and the discovery of biopsy sites for treatment optimization, resulting in a more expansive view of the effects of ^18^F-FDG-PET/CT.

The majority of scans in our cohort (68.1%) showed no scan-induced changes, as assessed in this study. However, as Bossuyt et al. [19] reported, the clinical utility of a test extends beyond its medical applications to include psychosocial factors. For instance, while the scans in our cohort did not lead to changes in clinical management, they may still have provided reassurance to patients undergoing surveillance for recurrence.

### Incidental finding

Recognizing that ^18^F-FDG-PET/CT can detect incidental findings [24,29], it is essential to consider their clinical impact. In a cohort of high-risk primary BC patients undergoing ^18^F-FDG-PET as part of initial work-up, up to one-third of the scans revealed incidental findings [24]. In our study, 4.0% (6 out of 151) of first-time ^18^F-FDG-PET scans (per-patient analysis) resulted in incidental findings. The variation in proportions may be attributed to different BC scenarios and variations in previous imaging procedures. Moreover, what remains uncertain is whether upstaging and the management adjustments prompted by ^18^F-FDG PET/CT could occasionally result in undertreatment. For example, altering the treatment approach could impact systemic treatment, local surgery, or the extension of radiation treatment fields. Consequently, this may not necessarily be beneficial for every individual patient. Clinicians must balance the advantages of precise staging with the potential risks. Multidisciplinary discussions are imperative to inform optimal, personalized treatment decisions.

### Treatment evaluation

Metabolic changes might predict treatment response earlier than structural changes and ^18^F-FDG-PET/CT in BC holds potential as a useful imaging modality for treatment response evaluation. Importantly, early identification of non-responders [30] enables re-evaluation of unbeneficial treatment regimens. Evaluating metabolic response in patients undergoing neoadjuvant therapy have shown promising results, e.g. in their meta-analysis, Tian et al. [28] included 22 studies evaluating the accuracy of ^18^F-FDG-PET/CT in assessing treatment response to neoadjuvant chemotherapy, where the results showed a pooled sensitivity of 0.82 for predicting pathological response early on during neoadjuvant chemotherapy. Moreover, other modalities are being compared to ^18^F-FDG-PET/CT for assessment of treatment response in this setting; in a study by Choi et al. [26], compared to MRI, changes in ^18^F-FDG metabolism had a higher discriminative performance (responders vs. non-responders), with a sensitivity of 0.83.

In the metastatic BC setting, ^18^F-FDG-PET/CT has demonstrated higher accuracy for response evaluation than conventional imaging techniques such as CT and bone scintigraphy [31]. Notably, ^18^F-FDG-PET/CT demonstrates greater sensitivity in detecting both progressive and regressive disease, whereas conventional imaging tends to classify disease as stable more frequently [25]. Moreover, a prospective observational study by Vogsen et al. (*N* = 87) demonstrated that ^18^F-FDG-PET/CT was a better predictor of progression-free and disease-specific survival than CT [32].

Treatment evaluation using ^18^F-FDG-PET/CT in both neoadjuvant and metastatic BC settings would be a clinically important field to explore in future studies, preferably multicenter randomized clinical trials with endpoints including patients’ survival and quality of life.

### Strengths and limitations

The consecutive cohort comprised BC patients who underwent ^18^F-FDG-PET/CT scans, resulting in a diverse population with varying disease stages, including metastatic disease, and various histopathological subtypes, reflecting the current clinical landscape. This differs from previous studies that focused on a more selected group of patients [3,22–24,33]. The number of scans in this study was relatively large compared to that in previous studies [22,23,33]; although not sufficient for subgroup analyses. Moreover, we presented both per-scan and per-patient data. Our study offers a comprehensive examination of the impact of scans on clinical management, contrasting with previous studies [3,23,24] that have regarded these changes as more general in nature. However, psychosocial factors in addition to health economical aspects were out of scope of this study.

As a retrospective, bi-center, observational study, the very nature of the study presents certain limitations. Ideally, patient outcomes should be evaluated through a randomized, prospective study design. Inclusion bias resulting from the selection of a particular group of patients who undergo ^18^F-FDG-PET/CT examinations is unavoidable and may have a more significant impact on early-stage patients.

The included patients underwent varying numbers of scans, which could potentially introduce a bias toward an overestimation of scan-induced changes. This is because patients with multiple scans may have more ^18^F-FDG-avid BC, and thus better visualized using ^18^F-FDG-PET/CT compared to patients with single scans. However, as demonstrated in the per-patient analyses, considering single scans for each patient led to a higher number of both restaging and changes in clinical management.

The standard practice at both study sites was to perform a diagnostic CT only when none had been done in the preceding 6–8 weeks, we therefore believe that the impact of ^18^F-FDG-PET on clinical management is consistent regardless of the CT quality.

### Future aspects

While ^18^F-FDG is the most clinically used PET tracer in BC imaging, there are many other radiotracers in clinical use and under evaluation, which would be interesting to investigate. In addition, it would be interesting to explore the clinical consequences of whole-body parametric imaging of ^18^F-FDG-PET as well as that of PET/MRI. Treatment evaluation using ^18^F-FDG-PET/CT in both neoadjuvant and metastatic BC settings is a clinically important field to explore in future studies.

## Conclusion

With indications for referral mirroring the present clinical landscape of individuals with BC stages I–IV, our research has demonstrated that ^18^F-FDG-PET/CT is a valuable tool in a wide range of clinical settings. We observed ^18^F-FDG-PET/CT-induced changes in clinical management in almost one-third of the cases, with the highest rates in patients undergoing initial work-up. However, in the recurrence group, more than every fourth scan led to a change from curative/no treatment to palliative treatment. These findings can help to further explore the use of ^18^F-FDG-PET/CT in BC. Prospective studies examining the clinical utility and patient outcomes are warranted.

## Supplementary Material

^18^F-FDG-PET/CT in breast cancer imaging: Restaging and Implications for treatment decisions in a clinical practice setting

## Data Availability

The datasets generated and/or analyzed in the current study are not publicly available because of privacy and ethical restrictions. However, should a researcher be interested in the data, they are welcome to contact the corresponding author.
