# Supplementary material for: 18F-FDG-PET/CT in breast cancer imaging: Restaging and Implications for treatment decisions in a clinical practice setting
Source: Acta Oncol. 2024 Aug 11;63:40003. doi: 10.2340/1651-226X.2024.40003 (PMC11332462; doi:10.2340/1651-226X.2024.40003)
Supplement: 18F-FDG-PET/CT in breast cancer imaging: Restaging and Implications for treatment decisions in a clinical practice setting [file AO-63-40003-s1.pdf]

Supplementary material has been published as submitted. It has not been copyedited, or typeset by Acta Oncologica

Supplementary Material 1. Indications for ^{18}F-FDG-PET/CT in breast cancer according to guidelines

	Swedish national guidelines [16]	ACR Appropriateness Criteria [14]	NCCN® Guidelines Version 3.2024 [13]	ESMO [17, 18]
**Initial workup/staging**	To rule out other primary tumors in patients with occult breast cancer.<br>Results from CT, MRI and ultrasound are inconclusive or unclear.	Locoregional or distant disease evaluation in patients with newly diagnosed breast cancer, clinical stage IIB-III (usually appropriate)<br>Response-assessment after completion of neoadjuvant chemotherapy in suspected metastatic disease (usually appropriate)<br>Evaluation of axilla in clinically node positive prior to neoadjuvant chemotherapy (may be appropriate)<br>Axilla imaging in newly diagnosed breast cancer >2 cm, clinical node positive, diagnostic mammography or DBT performed before treatment (may be appropriate)<br>Axilla imaging in newly diagnosed local recurrent breast cancer, diagnostic mammography or DBT performed (may be appropriate	Routine use of ^{18}F-FDG-PET/CT is not recommended in the staging of clinical stage I, II, or operable III (T3, N1) breast cancer.<br>In early breast cancer (T1-T3) with ≥N2, when consider imaging for systemic staging, ^{18}F-FDG-PET/CT is optional.<br>Optional in initial work up inflammatory breast cancer.<br>Optional in workup prior to preoperative systemic therapy, especially for stage III and invasive ductal histology.	Conventional methods (CT, ultrasound, MRI) are inconclusive<br>Staging in high-risk patients<br>Exclusion of other primary tumor in occult breast cancer<br>^{18}F-FDG-PET/CT may be useful when conventional methods are inconclusive.<br>^{18}F-FDG-PET/CT can also replace traditional imaging for staging in high-risk patients.<br>Management of occult breast cancer: Routine diagnosis, apart from standard breast and axillary imaging, requires breast MRI and PET-CT (to exclude another primary tumor site).
**Metastatic breast cancer**	Patients exhibiting specific symptoms, e.g., bone pain.<br>Results from CT-scans are inconclusive or unclear.<br>Mapping out distant metastases, enabling radical, local treatment in cases with singular distant metastasis.	Breast cancer, metastatic disease suspected, staging, initial imaging (usually appropriate)	In certain circumstances, ^{18}F-FDG-PET/CT in Stage IV (M1) or Recurrent disease.	Replacement of CT and bone scans as part of staging and risk assessment<br>Monitoring of bone-only/-predominant metastases<br>^{18}F-FDG-PET/CT may be used instead of CT and bone scans.<br>^{18}F-FDG-PET/CT might provide earlier guidance in monitoring bone-only/predominant metastases.

**Recurrence**	Ruling out distant metastatic recurrence, thus enabling locoregional treatment	Breast cancer (NST and ILC), any clinical stage at original presentation, distant recurrence suspected	In certain circumstances, ^{18}F-FDG-PET/CT in Stage IV (M1) or Recurrent disease.	

Abbreviations: ACR, American College of Radiology; ASCO, American Society of Clinical Oncology; ESMO, European Society for Medical Oncology; NCCN, National Comprehensive Cancer Network; CT, computed tomography; MRI, Magnetic resonance imaging; ^{18}F-FDG-PET/CT, ^{18}F-fluorodeoxyglucose – Positron emission tomography/Computed tomography; NST, Non-specific type; ILC, Invasive lobular cancer; DBT, Digital breast tomosynthesis

References:

13. NCCN Clinical Practice Guidelines in Oncology (NCCN Guidelines®) for Guideline Breast Cancer Version 3.2024 Arr. National Comprehensive Cancer Network.

14. Radiology ACo. ACR Appropriateness Criteria® Reston, Virginia: American College of Radiology; Available from: https://www.acr.org/Clinical-Resources/ACR-Appropriateness-Criteria

16. Regionala cancercentrum i samverkan, Nationellt vårdprogram för bröstcancer version 4.3. Stockholm, Sweden [Nov 22, 2023]; Available from: https://kunskapsbanken.cancercentrum.se/diagnoser/brostcancer/vardprogram/

17. Cardoso F, Kyriakides S, Ohno S, Penault-Llorca F, Poortmans P, Rubio IT, et al. Early breast cancer: ESMO Clinical Practice Guidelines for diagnosis, treatment and follow-updagger. Ann Oncol. 2019 Aug 1;30(8):1194-220. PMID: 31161190. doi: 10.1093/annonc/mdz173.

18. Gennari A, Andre F, Barrios CH, Cortes J, de Azambuja E, DeMichele A, et al. ESMO Clinical Practice Guideline for the diagnosis, staging and treatment of patients with metastatic breast cancer. Ann Oncol. 2021 Dec;32(12):1475-95. PMID: 34678411. doi: 10.1016/j.annonc.2021.09.019.

Supplementary Material 2. Cohort characteristics pre-scan: Patients' and tumor characteristics at time of referral to ^{18}F-FDG-PET/CT, **per-patient analysis**.

Patients, n = 151		Group A n = 35	Group B n = 76	Group C n = 40	*p* value
Age at first scan (years)	median (IQR)	60.5 (48.0-69.5)	62.0 (53.0-73.0)	59.0 (47.0-72.0)	0.598
BMI (kg/m^2)	median (IQR)	25.2 (22.1-28.2)	26.3 (23.8-29.3)	25.9 (22.0-29.8)	0.210
	missing (n)	2	8	1	
Blood glucose (mmol/L)	median (IQR)	5.6 (5.1-6.3)	5.5 (5.1-6.0)	5.6 (5.0-6.1)	0.916
	missing (n)	3	11	2	
Estrogen receptor status	positive	25 (71.4%)	57 (75.0%)	24 (60.0%)	0.763
	negative	9 (25.7%)	17 (22.4%)	10 (25.0%)	
	missing	1 (2.9%)	2 (2.6%)	6 (15.0%)	
Progesterone receptor status	positive	24 (68.6%)	45 (59.2%)	21 (52.5%)	0.512
	negative	9 (25.7%)	28 (36.8%)	13 (32.5%)	
	missing	2 (5.7%)	3 (3.9%)	6 (15.0%)	
HER2 status	positive	6 (17.1%)	10 (13.2%)	3 (7.5%)	0.528
	negative	26 (74.3%)	63 (82.9%)	30 (75.0%)	
	missing	3 (8.6%)	3 (3.9%)	7 (17.5%)	
Ki67	>20% (high)	27 (77.1%)	41 (53.9%)	19 (47.5%)	0.249
	<=20% (low)	5 (14.3%)	19 (25.0%)	7 (17.5%)	
	missing	3 (8.6%)	16 (21.1%)	14 (35.0%)	
Pre-scan TNM stage	No evidence of disease	0	7 (9.2%)	32 (80.0%)	<0.001*
	I	0	1 (1.3%)	0	
	II	17 (48.6%)	5 (6.6%)	0	
	III	16 (45.7%)	26 (34.2%)	4 (10.0%)	
	IV	2 (5.7%)	37 (48.7%)	4 (10.0%)	

Chi square test for categorical variables and Kruskal Wallis test for continuous variables.

Abbreviations: BMI, body mass index; ^{18}F-FDG-PET/CT, ^{18}F-fluorodeoxyglucose – Positron emission tomography/Computed tomography; HER2 = Human Epidermal growth factor Receptor 2; TNM, tumor node metastases

*$p < 0.05$

Supplementary Material 3. Histologic subtype of cohort, per-scan analysis, n (%)

Total<br>376 scans	Group A<br>35 scans	Group B<br>291 scans	Group C<br>50 scans	*p* value
NST	22 (62.9%)	207 (71.1%)	38 (76.0%)	0.005*
ILC	7 (20.0%)	60 (20.6%)	6 (12.0%)	
Mixed (NST + ILC)	1 (2.9%)	8 (2.7%)	0	
DCIS only	1 (2.9%)	5 (1.7%)	4 (8.0%)	
LCIS only	0	1 (0.3%)	0	
Other	2 (5.7%)	0	0	
Missing	2	10	2	

Abbreviations: NST, non-specific type; ILC, invasive lobular cancer; DCIS, ductal carcinoma in situ; LCIS, lobular carcinoma in situ.

*p <0.05, Chi Square test

Supplementary Material 4. Cohort characteristics, pre-scan: Previous/ongoing oncological treatment, and breast cancer related surgeries at time of referral to ^{18}F-FDG-PET/CT, **per-patients analysis (first scan)**, n (%)

Number of patients		Group A,	Group B	Group C	*p* value
Total, n = 151		n = 35	n = 76,	n = 40	
Previous neoadjuvant chemotherapy	yes	2 (5.7%)	26 (34.2%)	9 (22.5%)	0.005*
	no	33 (94.3%)	50 (65.8%)	30 (75.0%)	
	missing	0	0	1 (2.5%)	
Previous neoadjuvant HER2-targeted therapy	yes	1 (2.9%)	4 (5.3%)	2 (5.0%)	0.840
	no	34 (97.1%)	71 (93.4%)	37 (92.5%)	
	missing	0	1 (1.3%)	1 (2.5%)	
Previous adjuvant chemotherapy	yes	0	17 (22.4%)	23 (57.5%)	<0.001*
	no	35	58 (76.3%)	16 (40.0%)	
	missing	0	1 (1.3%)	1 (2.5%)	
Previous adjuvant HER2-targeted therapy	yes	0	2 (2.6%)	3 (7.5%)	0.166
	no	35	73 (96.1%)	36 (90.0%)	
	missing	0	1 (1.3%)	1 (2.5%)	
Previous adjuvant endocrine therapy	yes	2 (5.7%)	28 (36.8%)	8 (20.0%)	0.002*
	no	33 (94.3%)	48 (63.2%)	31 (77.5%)	
	missing	0	0	1 (2.5%)	
Previous adjuvant radiation therapy	yes	1 (2.9%)	31 (40.8%)	26 (65.0%)	<0.001*
	no	34 (97.1%)	45 (59.2%)	13 (32.5%)	
	missing	0	0	1 (2.5%)	
Previous palliative chemotherapy	yes	0	18 (23.7%)	0	<0.001*
	no	35	57 (75.0%)	39 (97.5%)	
	missing	0	1 (1.3%)	1 (2.5%)	
Previous palliative HER2-targeted therapy	yes	0	1 (1.3%)	0	0.609
	no	35	74 (97.4%)	39 (97.5%)	
	missing	0	1 (1.3%)	1 (2.5%)	
Previous palliative endocrine therapy	yes	0	12 (15.8%)	0	0.002*
	no	35	63 (82.9%)	39 (97.5%)	
	missing	0	1 (1.3%)	1 (2.5%)	
Previous palliative radiation therapy	yes	0	10 (13.2%)	0	0.005*
	no	35	65 (85.5%)	39 (97.5%)	
	missing	0	1 (1.3%)	1 (2.5%)	
Previous CDK 4/6-inhibitors therapy	yes	0	3 (3.9%)	0	0.216

	no	35	71 (93.4%)	39 (97.5%)	
	missing	0	2 (2.6%)	1 (2.5%)	
Ongoing neoadjuvant chemotherapy	yes	3 (8.6%)	5 (6.6%)	0	0.197
	no	32 (91.4%)	70 (92.1%)	40	
	missing	0	1 (1.3%)	0	
Ongoing neoadjuvant HER2-targeted therapy	yes	1 (2.9%)	1 (1.3%)	0	0.560
	no	34 (97.1%)	74 (97.4%)	40	
	missing	0	1 (1.3%)	0	
Ongoing adjuvant chemotherapy	yes	0	3 (3.9%)	0	0.216
	no	35	72 (94.7%)	40	
	missing	0	1 (1.3%)	0	
Ongoing adjuvant HER2-targeted therapy	yes	0	0	0	N/A**
	no	35	76 (100%)	40	
	missing	0	0	0	
Ongoing adjuvant endocrine therapy	yes	0	7 (9.2%)	14 (35.0%)	<0.001*
	no	35	68 (89.5%)	26 (65.0%)	
	missing	0	1 (1.3%)	0	
Ongoing adjuvant radiotherapy	yes	0	1 (1.3%)	1 (2.5%)	0.642
	no	35	74 (97.4%)	39 (97.5%)	
	missing	0	1 (1.3%)	0	
Ongoing palliative chemotherapy	yes	0	16 (21.1%)	1 (2.5%)	<0.001*
	no	35	59 (77.6%)	39 (97.5%)	
	missing	0	1 (1.3%)	0	
Ongoing palliative HER2-targeted therapy	yes	1 (2.9%)	5 (6.6%)	0	0.204
	no	34 (97.1%)	70 (92.1%)	40	
	missing	0	1 (1.3%)	0	
Ongoing palliative endocrine therapy	yes	2 (5.7%)	22 (28.9%)	1 (2.5%)	<0.001*
	no	33 (94.3%)	53 (69.7%)	39 (97.5%)	
	missing	0	1 (1.3%)	0	
Ongoing palliative radiotherapy	yes	0	1 (1.3%)	0	0.604
	no	35	74 (97.4%)	40	
	missing	0	1 (1.3%)	0	
Ongoing CDK4/6-inhibitor therapy	yes	0	12 (15.8%)	0	0.001*
	no	35	63 (82.9%)	40	
	missing	0	1 (1.3%)	0	

Axillary surgery	no axillary surgery	32 (91.4%)	19 (25.0%)	6 (15.0%)	<0.001*
	SLNB only	2 (5.7%)	20 (26.3%)	18 (45.0%)	
	ALND only	0	31 (40.8%)	10 (25.0%)	
	SLNB + ALND	1 (2.9%)0	6 (7.9%)0	6 (15.0%)0	
Breast surgery method	no breast surgery	33 (94.3%)	13 (17.1%)	3 (7.5%)	<0.001*
	sector	1 (2.9%)	26 (34.2%)	16 (40.0%)	
	mastectomy	1 (2.9%)	37 (48.7%)	21 (52.5%)	
	missing	0	0	0	

Categorical variables summarized as counts and percentages and continuous variables as medians and interquartile ranges (IQRs).

Abbreviations: ALND, axillary lymph node dissection; ^{18}F-FDG-PET/CT, ^{18}F-fluorodeoxyglucose – Positron emission tomography/Computed tomography; HER2 = Human Epidermal growth factor Receptor 2; SLNB, sentinel lymph node biopsy

*p < 0.05
**No statistics are computed because variable is constant.

Supplementary Material 5. Change in stage, difference between stage prior to and after ^{18}F-FDG-PET/CT-scan, stratified by stage prior to scan.

Change in stage (difference between stage pre-scan and post-scan)	-4	-3	-2	-1	0	1	2	3	4	Total restaging
No evidence of disease (56 scans)	8	4	0	3	41	0	0	0	0	15 (26.8%)
I (3 scans)	0	1	0	0	2	0	0	0	0	1 (33.3%)
II (25 scans)	0	0	6	5	14	0	0	0	0	11 (44.0%)
III (103 scans)	0	0	0	15	82	3	0	3	0	21 (20.4%)
IV (189 scans)	0	0	0	0	187	2	0	0	0	2 (1.1%)
Total (376 scans)	8	5	6	23	326	5	0	3	0	50 (13.3%)

Supplementary Material 6. TNM stage prior and after ^{18}F-FDG-PET/CT scan, **per-patients analysis**, n (%)

Group	Stage	Before ^{18}F-FDG-PET/CT	After ^{18}F-FDG-PET/CT	*p* value
A, Primary staging (35 scans)	I	0	0	<0.001*
	II	17 (48.6%)	7 (20.0%)	
	III	16 (45.7%)	14 (40.0%)	
	IV	2 (5.7%)	14 (40.0%)	
B, Response evaluation (76 scans)	NED	7 (9.2%)	8 (10.5%)	0.378
	I	1 (1.3%)	2 (2.6%)	
	II	5 (6.6%)	4 (5.3%)	
	III	26 (34.2%)	19 (25.0%)	
	IV	37 (48.7%)	43 (56.6%)	
C, Recurrence (40 scans)	NED	32 (80.0%)	21 (52.5%)	<0.002*
	I	0	1 (2.5%)	
	II	0	0	
	III	4 (10.0%)	5 (12.5%)	
	IV	4 (10.0%)	13 (32.5%)	

Abbreviations: TNM, tumor node metastases; ^{18}F-FDG-PET/CT, ^{18}F-fluorodeoxyglucose – Positron emission tomography/Computed tomography, NED, no evidence of disease

Wilcoxon Signed Ranks Test, *$p < 0.05$

Supplementary Material 7. Change in stage, difference between stage prior to and after ^{18}F-FDG-PET/CT-scan, stratified by indication, **per-patient analysis.**

Change in stage (difference between stage pre-scan and post-scan)	-4	-3	-2	-1	0	1	2	3	4	Total restaging
A, Primary staging (35 scans)	0	0	6	10	19	0	0	0	0	16 (45.7%)
B, Response evaluation (76 scans)	0	0	0	9	64	1	0	2	0	12 (15.8%)
C, Recurrence (40 scans)	8	2	0	2	28	0	0	0	0	12 (30.0%)
Total, 151 scans	8	2	6	21	111	1	0	2	0	40 (26.5%)

Supplementary Material 8. Change in stage, difference between stage prior to and after

^{18}F-FDG-PET/CT-scan, stratified by stage prior to scan, **per-patient analysis.**

Change in stage (difference between stage pre-scan and post-scan)	-4	-3	-2	-1	0	1	2	3	4	Total restaging
No evidence of disease (39 scans)	8	2	0	2	27	0	0	0	0	12 (30.8%)
I (1 scans)	0	0	0	0	1	0	0	0	0	0
II (22 scans)	0	0	6	5	11	0	0	0	0	11 (50.0%)
III (46 scans)	0	0	0	14	30	0	0	2	0	16 (34.8%)
IV (43 scans)	0	0	0	0	42	1	0	0	0	1 (2.3%)
Total (151 scans)	8	2	6	21	111	1	0	2	0	40 (26.5%)

A

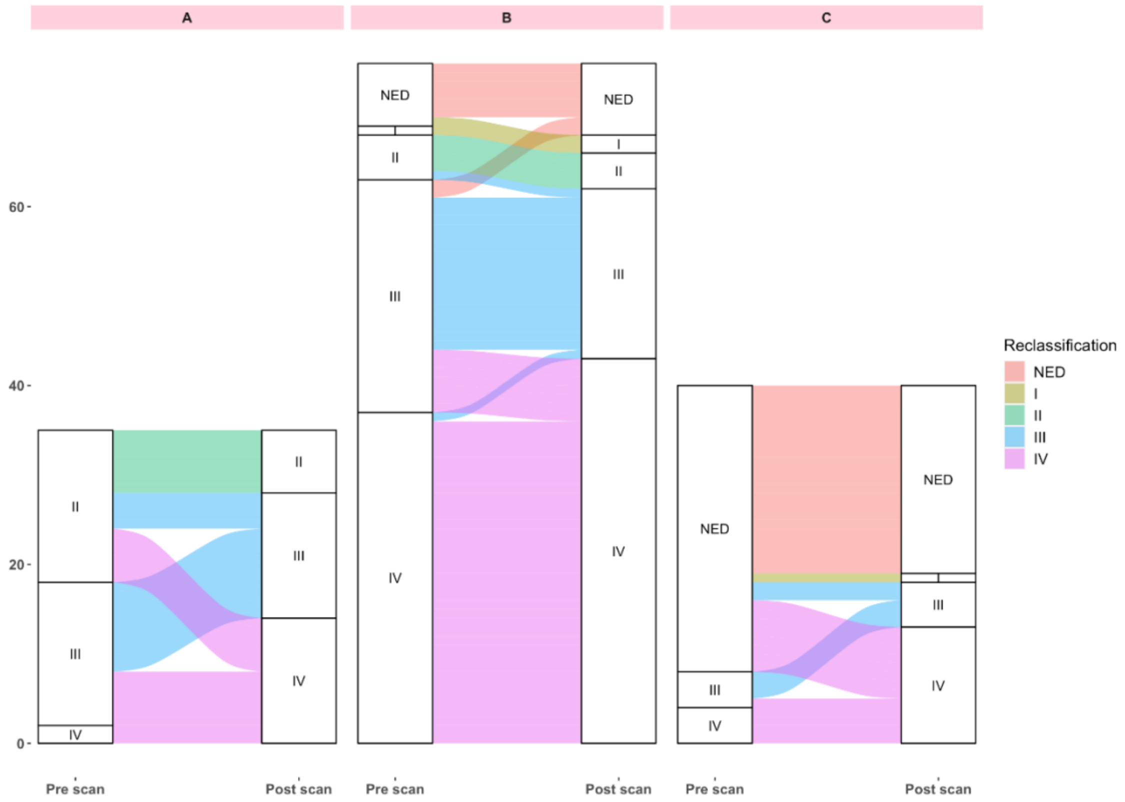

B

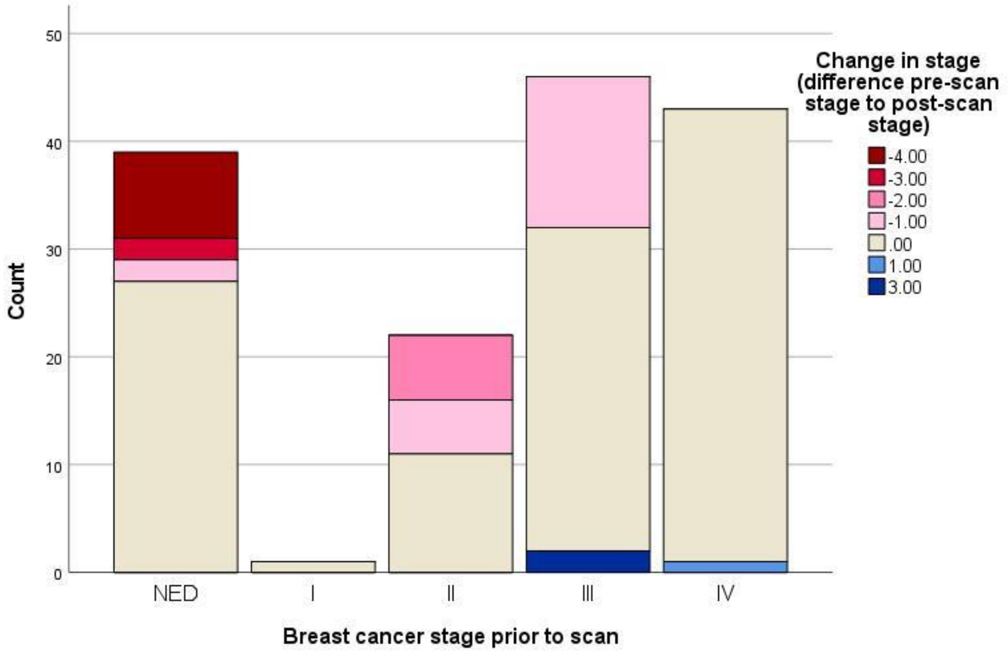

Supplementary Material 9.

A) Sankey diagram visualizing changes in breast cancer stage in each group (A-C), **per-patient analysis**.

B) Restaging according to stage prior to scan. -4 to -1 indicates upstaging, 0 equals no change in stage, and 1 -3 indicates downstaging, **per-patient analysis**.

Supplementary Material 10. ^{18}F-FDG-PET/CT scan-induced changes in clinical management, **per-patient analysis**

Type of change	No change	Minor change						Major change								Total
		1	2	3	4	5	total	1	2	3	4	5	6	7	total	
A, Primary staging (35 scans)	15	1	0	0	2	0	3	0	15	0	0	0	2	0	17	20 (57.1%)
B, Response evaluation (76 scans)	51	0	8	1	0	1	10	1	8	0	1	1	2	2	15	25 (32.9%)
C, Recurrence (40 scans)	24	0	0	1	0	0	1	0	6	0	9	0	0	0	15	16 (40.0%)
Total, 151 scans	90	2	42	6	2	2	14	3	30	1	12	6	4	10	47	61 (40.4%)

Minor change: 1 Modified radiotherapy; 2 Modified systemic treatment; 3 Biopsy to further optimize clinical management; 4 Avoidance of unnecessary diagnostics; 5 Modified surgical plan

Major change: 1 Downstaging and change from palliative to curative; 2 Upstaging and change from curative to palliative; 3 Complete remission, change from treatment to non-treatment; 4 Change from non-treatment to treatment; 5 Secondary findings on ^{18}F-FDG-PET/CT-scan affecting clinical management (e.g. other malignancy/pathology); 6 ^{18}F-FDG-PET/CT-scan guides treatment plan; 7 Addition/exclusion of surgery and/or radiotherapy to systemic treatment
